# How Context Shapes Person‐Centred Fundamental Care Through Nurse–Patient Relationships: Validation of the FoC Intelligence Modelling Tool and Predictive Pathway Analysis

**DOI:** 10.1111/jan.70335

**Published:** 2025-11-11

**Authors:** Regina Allande‐Cussó, Maria Alejandra Pinero de Plaza, Alison Kitson, Rebecca Feo, Tiffany Conroy, Ana María Porcel‐Gálvez

**Affiliations:** ^1^ Flinders University, College of Nursing and Health Sciences Adelaide South Australia Australia; ^2^ Faculty of Nursing, Physiotherapy and Podiatry University of Seville Seville Spain; ^3^ Research Group PAIDI‐CTS 1050 Complex Care Chronicity and Health Outcomes of the Institute of Biomedicine of Seville Seville Spain; ^4^ Adelaide Medical School, The University of Adelaide South Australia Australia; ^5^ Queensland University of Technology Brisbane Australia; ^6^ Aalborg University Aalborg Denmark; ^7^ University College Dublin Belfield Dublin Ireland; ^8^ University of Sydney, Sydney, New South Wales Australia

**Keywords:** fundamentals of care, nurse–patient relationship, patient experience, patient‐centred care, psychometrics, structural equation modelling

## Abstract

**Background:**

The Fundamentals of Care (FoC) Framework emphasises that care quality depends not only on clinical tasks but also on interpersonal relationships and the organisational context in which care is delivered. Although patient‐reported outcome and experience measures (PROMs and PREMs) have gained relevance in capturing these aspects, tools based on the FoC Framework remain limited in non‐English‐speaking settings.

**Objectives:**

To psychometrically validate the Spanish version of the FoC Intelligence Modelling Tool (FoC‐IMT) and explore predictive relationships among the FoC dimensions: Context, Relationship and Integration of Care.

**Methods:**

A cross‐sectional study was conducted with 1053 hospitalised patients in southern Spain. Exploratory and confirmatory factor analyses (EFA and CFA) were performed, alongside a mediation analysis using partial least squares structural equation modelling (PLS‐SEM) to examine directional relationships among constructs.

**Results:**

EFA and CFA supported a two‐factor model—Context and Integration of Care—with excellent internal consistency (Cronbach's *α* and McDonald's *ω* = 0.97). CFA showed a moderate correlation between these factors. However, PLS‐SEM mediation analysis revealed a directional model in which Context influences Relationship (*β* = 0.39), which in turn predicts Integration of Care (*β* = 0.89). Although embedded within Integration under CFA, the Relationship showed independent predictive power in PLS‐SEM, validating its conceptual importance. This aligns with the foundational assumption of the FoC Framework: that caregiving quality is shaped not only by tasks or procedures but by the broader environment and interpersonal relationships in which care occurs.

**Conclusions:**

The Spanish FoC‐IMT Tool is a valid, reliable instrument for assessing person‐centred care. The predictive model highlights the pivotal role of therapeutic relationships in delivering integrated, high‐quality care.

**Patient or Public Contribution:**

Hospitalised patients contributed directly by responding to the FoC‐IMT survey, thereby shaping the psychometric validation and predictive model. Patients were not involved in the study design, conduct or manuscript preparation.

## 1 Introduction

In the contemporary, increasingly complex healthcare landscape, evaluating care quality and patient safety requires more than traditional clinical indicators. Incorporating patient perspectives and reported outcomes helps reveal care dimensions often overlooked by biomedical models, such as therapeutic relationships or care environment quality. To capture these aspects, specific methodologies have been developed (Falade et al. 2024), primarily patient‐reported outcome measures (PROMs) and patient‐reported experience measures (PREMs). PROMs assess health status, functionality and quality of life from the patient's viewpoint, while PREMs address experiences related to communication, service responsiveness and care settings (Zabaleta‐del‐Olmo and González‐del‐Río 2021). Such approaches are particularly relevant in high‐vulnerability or high‐dependency healthcare contexts, where shared decision‐making and aligning care with patient preferences are crucial for safety and effectiveness (Kynoch et al. 2021).

The integration of PROMs and PREMs into routine clinical practice has gained international traction to foster patient‐centred care and inform quality improvement initiatives (Pennucci et al. 2019). For instance, a systematic review highlighted that the use of PREMs can enhance patient engagement and facilitate the co‐production of care, leading to improved health outcomes and patient satisfaction (Shunmuga Sundaram et al. 2022). Furthermore, the international momentum reflects a growing consensus on the value of incorporating patient feedback into healthcare evaluation and policymaking (Flott et al. 2017), especially in Nursing Science (Sim et al. 2018). The relationship between patient experiences and outcomes is complex and multifaceted (Schwartz et al. 2021). Studies have shown that positive patient experiences are often associated with better health outcomes, including improved adherence to treatment regimens and enhanced recovery processes. For example, research examining elective surgical procedures found a weak positive association between patient‐reported experiences and health outcomes, suggesting that patients who report better experiences may also achieve better clinical results (Black et al. 2014). These findings highlight the importance of measuring both experiences and outcomes to gain a holistic understanding of care quality and identify areas for improvement (Kuluski et al. 2017).

Ensuring the quality and safety of healthcare necessitates the active involvement of all stakeholders, particularly patients (Sarkhosh et al. 2022). Patient engagement is recognised as a critical component in the design and delivery of healthcare services (Bombard et al. 2018). By actively involving patients in their care, nurses can better understand patient needs, preferences and values, leading to more personalised and effective care plans (Jeffs et al. 2016). Moreover, patient involvement in quality improvement initiatives can provide unique insights that may not be apparent to nurses alone (Bergerum et al. 2020). This collaborative approach fosters a culture of transparency and mutual respect, ultimately contributing to enhanced care quality and patient satisfaction (Vaismoradi et al. 2015). In addition, the Picker Institute Europe has long established a framework for person‐centred care based on eight principles that emphasise patient engagement to improve patient autonomy, communication, emotional support and continuity of care. Although these principles have been integrated into policies and professional standards in countries such as the United Kingdom, Canada, Australia and Spain, international research has revealed variability in their implementation, particularly between service users' expectations and professional priorities (Hyde and Hardy 2021).

As a discipline, Nursing places particular emphasis on the relational and person‐centred dimensions of care, which are central to understanding patient experience (Ross et al. 2015). This contribution to the broader debate on person‐centred care builds on a well‐established foundation in nursing scholarship, particularly the recognised importance of the nurse–patient relationship within the Fundamentals of Care (FoC) Framework (Feo et al. 2017). This emphasis has been consistently articulated through the global advocacy work of the International Learning Collaborative (ILC) (International Learning Collaborative 2025). Since 2010, the ILC has played a key role in advancing this agenda by bringing together international leaders in nursing education, research and clinical practice to improve the delivery of fundamental care. Fundamental care describes the universal needs of people, both well and with health challenges, that need to be met for them to maintain health and wellness (Kitson et al. 2013). The FoC Framework was co‐designed by members of the ILC along with service users and stands out for its comprehensive approach to understanding and enhancing care delivery from the patient's perspective (Feo et al. 2018; Kitson et al. 2013). The FoC Framework posits that high‐quality care emerges through the dynamic interplay of three core dimensions: the establishment of a therapeutic relationship between care provider and patient; the integration of physical, psychosocial and relational care needs; and the influence of the context of care, including organisational and systemic factors (Kitson 2018). This framework emphasises that care provision is inherently relational and contextual, rather than solely procedural or task‐oriented (Kitson et al. 2014). Moreover, it supports a person‐centred perspective by advocating for the active involvement of patients in care processes, recognising them as partners in the co‐production of health outcomes (Kitson 2018).

Despite the emerging theoretical and practical relevance of the FoC Framework, tools that effectively operationalise its components remain scarce, particularly in non‐English‐speaking contexts (Palese et al. 2021). In an increasingly globalised and multicultural health landscape, it is critical to ensure that conceptual frameworks and measurement instruments are both linguistically and culturally valid (Valdez et al. 2021). Recent international efforts have begun to validate instruments derived from the FoC Framework in various contexts, such as the *FoC Intelligence Modelling Tool (FoC‐IMT)*, which captures the patient's experience of care by assessing elements related to context, relationship and integration (Pinero de Plaza et al. 2025). This instrument has demonstrated promising psychometric properties in its original English and Spanish versions in a pilot study; however, its application and validation in a larger Spanish‐speaking sample remain underexplored (Pinero de Plaza et al. 2025).

Spain, like many other countries, is currently navigating challenges related to an ageing population, rising multimorbidity and increased demands for hospital‐based care (Spijker and Rentería 2023). These trends necessitate robust, evidence‐informed tools that can provide accurate assessments of care quality from the patient's viewpoint. Particularly in southern Spain, where regional variations in health service delivery and patient demographics are pronounced (Spijker and Rentería 2023), there is an urgent need for validated instruments that can inform local quality improvement strategies while contributing to broader international comparisons.

In this context, this study aims to assess the psychometric properties of the Spanish version of the *FoC‐IMT*, using exploratory and confirmatory factorial analyses, both based on covariance, in a population of hospitalised patients in southern Spain. Secondly, the study aims to design and validate a predictive model of the relationships between the core dimensions of the FoC Framework, using a partial least squares regression model that is based on variance. This predictive model design is based on the following hypotheses: considering the FoC Framework, the Context of Care has a positive impact on the Relationship dimension, which in turn facilitates the Integration of Care to take place. These associations are theoretically grounded and supported by preliminary findings from a previous pilot study that demonstrated the same significant links between these three dimensions (Pinero de Plaza et al. 2025).

## 2 Methods

### 2.1 Design

A descriptive, cross‐sectional study was carried out in two phases: an initial phase of psychometric validation and refinement of the tool, and a second phase of a predictive model design.

### 2.2 Population and Sample

#### 2.2.1 Population

The study was conducted in 2024 within the Andalusian Health Care System, located in southern Spain. Under the oversight of the regional government, a network of 34 public hospitals is organised into three categories based on their level of specialisation and the population size they serve: 9 primary hospitals with over 500 beds located in large metropolitan areas, 6 specialist hospitals with 200 to 500 beds in smaller metropolitan regions, and 19 tertiary hospitals with fewer than 200 beds situated in rural areas (Andalusian Health System 2021). The network of public hospitals reports an annual activity of 504,374 admissions, 3,489,333 patient days with an average stay of 6.34 days, and an occupancy rate of 62.77% (Andalusian Health System 2024). The hospitals offer comprehensive acute inpatient and outpatient services, along with emergency care facilities (Andalusian Health System 2022).

This study involved patients admitted to internal medicine units in eight tertiary hospitals: Hospital Universitario Virgen del Rocío (HUVR), Hospital Virgen Macarena (HUVM), Hospital Universitario Virgen de Valme (HUVV), Hospital Universitario San Juan de Dios–Bormujos (HUSJD), Hospital Universitario Reina Sofía (HURS), Hospital Universitario Puerta del Mar, Hospital San Carlos (HSC) y Hospital Universitario Virgen de las Nieves (HUVN).

#### 2.2.2 Sample

Sample size and sampling method were based on achieving representativeness. Representativeness was ensured by combining psychometric criteria (a minimum of 5 participants per item, requiring at least 205 cases for the 41‐item tool) with population‐based estimates (Beavers et al. 2013; Fox et al. 2009; Ramada et al. 2013). Based on official activity data of the eight hospitals involved (Andalusian Health System 2024), the total number of admissions was 1,346,628 (with a confidence level of 95% and an error margin of 3%), allowing for a 15% loss rate. Although the estimated sample size was 714 patients, the final recruited sample of 1053 exceeded these thresholds, thereby ensuring sufficient statistical power and representativeness.

Sampling was done consecutively according to the hospital type and the admission ward. Patients were selected on a random basis through their hospital admission on the sampling days. The inclusion criteria were patients older than 18 years and admitted to Internal Medicine wards. Individuals with severe health limitations affecting their ability to provide informed consent or reliable responses, and those who did not consent to participate, were excluded.

### 2.3 Recruitment and Training

The selection and recruitment of nurse interviewers were undertaken by the research project ‘Evaluación y desarrollo de la HUMANización del CUIDAdo del sistema sanitario público de Andalucía: atención a la fragilidad, prevención de eventos adversos, e impacto de la relación enfermera‐paciente en los resultados en salud (HUMANCUIDA) [Evaluation and Development of the HUMANisation of CARE within the Public Healthcare System of Andalusia: Addressing Frailty, Preventing Adverse Events and Assessing the Impact of the Nurse–Patient Relationship on Health Outcomes]’, funded by the Instituto de Salud Carlos III de Madrid and the Spanish Ministry of Health (PI 22/00373).

The research team for the HUMANCUIDA Project consisted of 18 nurses (research nursing team) and the coordinating team (two principal investigators). The research nursing team was selected in collaboration with the Nursing Directors of the hospitals participating in the study. The research nursing team was responsible for introducing the study to the clinical teams and ensuring ongoing oversight of its implementation within the participating units, under the training provided by the coordinating team. The participation of the research nursing team was voluntary, and no financial compensation was provided.

The recruitment of the patient sample was carried out through the subcontracting of a healthcare recruitment company specialising in health projects in Spain. This company was commissioned to execute the data collection phase of the study and to provide technical staff for the tasks contracted. Specifically, it selected 18 nurses with experience in hospital‐based data collection, who were financially compensated for their work, and they were external personnel of the research project team.

The coordinating team conducted two 5‐h training sessions with the research nursing team and the 18 individuals recruited from the healthcare recruitment company in December 2023. These sessions presented the HUMANCUIDA study in detail, outlining its objectives and focusing on the final survey structure and data collection procedure. Particular attention was paid to describing the purpose of each item and how to phrase the corresponding question.

### 2.4 Variables and Instruments

In this study, different types of variables were taken into consideration: socio‐demographic variables (sex, age, hospital and reason for hospital admission) and variables related to the questionnaire (the scores obtained in each item and the total score).

The FoC‐IMT was originally developed and piloted in English and Spanish to assess the core dimensions of the FoC Framework (Context, Relationship and Integration of Care) (Pinero de Plaza et al. 2025) (see Supporting Information). The Spanish version comprises 41 items scored on a 5‐point Likert scale, grouped into the three dimensions of the FoC Framework. In the present study, this version was subjected to full psychometric validation in a larger Spanish‐speaking patient sample.

### 2.5 Data Collection

The sample was selected over 6 months (January to June 2024). The survey was embedded in the LimeSurvey software under licence to ensure the secure storage of personal data. Data collection was therefore conducted via an online survey link, which the 18 recruited nurse interviewers completed on their tablets during interviews with patients. Before each interview, written informed consent to participate in the study was obtained from all participants without exception and physically delivered to the principal investigators at the end of the interview period.

The research nursing team participating in hospitals facilitated interviewers' access to the settings and provided personal protective equipment if necessary. Furthermore, no patient was interviewed or disturbed if their health condition on the sampling days was not considered minimal to guarantee their comfort or to hinder the care they required due to their hospital admission. This judgement was made jointly by the research nursing team at each participating hospital and the registered nurses on duty who held clinical responsibility for the patients.

### 2.6 Data Analysis

A univariate descriptive statistical analysis of the sample was carried out using SPSS v29 software (IBM Corporation 2019). Frequencies, percentages, means and standard deviations were calculated as appropriate for each variable to describe the sample profile.

To complement the analysis, both variance‐based (PLS‐SEM) and covariance‐based (CB‐SEM) structural equation modelling techniques were employed. PLS‐SEM, oriented towards prediction, aims to maximise the explained variance of the dependent constructs and is suitable for exploratory models and smaller sample sizes. In contrast, CB‐SEM seeks to reproduce the observed covariance matrix and is primarily used for theory confirmation, requiring stricter assumptions and larger samples. The combined use of both approaches allowed for robust estimation and validation of the proposed model (Hair et al. 2021).

As part of the CB‐SEM approach, exploratory factor analysis (EFA) and confirmatory factor analysis (CFA) were subsequently conducted to assess the dimensionality and validity of the measurement model. Firstly, an EFA was conducted using maximum likelihood estimation and varimax rotation to explore the factorial structure of the tool. Items with loadings below 0.5 were excluded from the final factor solution. Secondly, to examine criterion validity, a CFA was performed using AMOS v29 software (Arbuckle 2019). The following fit indices were used to evaluate the model fit: RMSEA (values ≤ 0.08 indicate a good fit), NFI (normalised fit index), CFI and TLI (values ≥ 0.90 indicate a good fit) (Hu and Bentler 1999). The reliability metrics tool, Cronbach's Alpha and McDonald's Omega, are detailed in the results section, providing a robust basis for the instruments' effectiveness in capturing accurate and reliable data. When the tau‐equivalence conditions (i.e., homogeneous covariance between true scores and item measurement errors) are not met, McDonald's Omega presents less risk of over‐ or underestimation of the reliability of the tool (Moral de la Rubia 2019). For the validation of the final factor model, the modification indices provided by the AMOS software were considered, which indicate which measurement errors of each item should be correlated. The aim was to achieve the best possible fit for the factor model, although the FoC Framework always served as the basis for identifying this fit and the meaning of these correlations between measurement errors (DeCastellarnau and Saris 2021). Furthermore, disregarding these correlations between measurement errors of the items, based on the modification indices suggested by the AMOS software, has negative implications for the reliability of the tool measured by Cronbach's alpha (Green and Hershberger 2000).

A mediation analysis was performed, using partial least squares structural equation modelling (PLS‐SEM), to explore and validate the predictive relationships between the three dimensions of the FoC Framework. In PLS‐SEM analyses, hypotheses are derived from a theoretical framework and tested accordingly. In addition, the dimensions should be named as composites, in accordance with the requirements of the PLS‐SEM approach (Hair et al. 2021). Thus, for this purpose, the consistent PLS‐SEM (PLSc‐SEM) algorithm performs a correction of reflective constructs' correlations, named composites in the PLS method, to make results consistent with a factor model (Dijkstra and Henseler 2015a, 2015b). Values Cronbach's alpha, Rho A and composite reliability (cut‐off values > 0.7) are calculated to assess internal consistency, while average variance extracted (AVE) is examined for convergent validity (cut‐off value > 0.5). Furthermore, the validity of items within each latent variable is assessed. The variance inflation factor (VIF) is examined to detect multicollinearity among items (< 3.3), and beta coefficients are calculated to quantify the strength and direction of the relationships between latent variables in the structural model. The algorithm also calculates the coefficient of determination *R* ^2^, which measures the amount of variance explained by the other composites above. In addition, the ADANCO software also determines the standardised root mean square residual values (SRMR) and the heterotrait‐monotrait (HTMT) coefficient to obtain the discriminant validity between composites (Henseler et al. 2015). In addition, the effect sizes (Cohen's *f* ^2^) of the relationships between the composites are calculated (cut‐off values > 0.15), and beta coefficients are calculated as the total effect of each composite correlated. The critical values of the fit parameters provided by the PLSc to be considered as optimal model are SRMR < 0.08; AVE ≥ 0.5; HTMT ≤ 0.85; VIF ≤ 5; Rho A, Cronbach's *α* and Composite Reliability > 0.7; and *f* ^2^ must be > 0.15 (Hair et al. 2019).

### 2.7 Ethical Considerations

Ethical approval was obtained from the Andalusian government's Ethics Committee (Approval Code 0840‐N‐22). All participants were informed of the purpose of the study and the possibility of participating on a voluntary, anonymous, and confidential basis before being interviewed.

Compliance with Spanish Organic Law 3/2018 of 5 December on the Protection of Personal Data and the Guarantee of Digital Rights (Gobierno de España‐Spanish Government 2018) and the principles of the Declaration of Helsinki (World Medical Association 2014) were ensured throughout the study. Regarding the processing of personal data, the corresponding registration was carried out with the University of Seville's Data Protection Department under the regulations stipulated by the Spanish Data Protection Agency (Spanish Government 2024).

## 3 Results

### 3.1 Characteristics of the Sample

The total sample consisted of 1053 inpatients. The results showed a mean age of 71.6 (SD = 16.4 years). Of the total sample, 52% were women and 48% were men. The percentages of inpatients from different hospitals participating are: 20% from HUVM, 18.3% from HUVR, 18.3% from HURS, 13.4% from HUVV, 8.1% from HUPM, 8% from HUSJD, 7.4% from HSC and 6.6% from HUVN. Concerning the reasons for admission, respiratory conditions were the most prevalent, with 26.7%. This was followed by cardiological conditions with 14.5% and processes related to fever and infections with 8%. Other reasons, such as abdominal, oncological, traumatological or neurological conditions, presented percentages lower than 8% of the sample.

### 3.2 Construct Validity

To determine the underlying structure of the measured construct, an EFA was conducted. The Kaiser‐Meyer‐Olkin test obtained a value of 0.98, and the Bartlett test of sphericity obtained a statistically significant value (*χ* ^2^ = 48,913.02; df = 741; *p* < 0.001). Using EFA, a dimensional matrix of 39 items and 2 factors explaining 72.5% of the variance was extracted. The two factors were named by the research team, considering the semantic content of the items that comprise them, as well as their alignment with the FoC Framework, to which all items are theoretically linked: Integration of Care and Context (Table 1). From a total of 41 items, 2 were eliminated (items 30 and 31 from the original list) because they had weights with a value lower than 0.4 or were redundant because they shared more variance between them than directly explained by a common factor (Lloret‐Segura et al. 2014). The reliability study yielded a Cronbach's *α* value = 0.97 and Omega de McDonald's value = 0.97 regarding the total scale; a Cronbach's *α* value = 0.98 for the Integration of Care factor and a Cronbach's *α* value = 0.96 for the Context factor were obtained.

**TABLE 1:** Exploratory factor analysis: Dimensional structure.

Items	Integration of care	Context
ITEM_1	0.750	
ITEM_2	0.751	
ITEM_3	0.754	
ITEM_4	0.748	
ITEM_5	0.756	
ITEM_6	0.747	
ITEM_7	0.800	
ITEM_8	0.822	
ITEM_9	0.815	
ITEM_10	0.856	
ITEM_11	0.835	
ITEM_12	0.833	
ITEM_13	0.837	
ITEM_14	0.833	
ITEM_15	0.812	
ITEM_16	0.675	
ITEM_17	0.801	
ITEM_18	0.834	
ITEM_19	0.832	
ITEM_20	0.834	
ITEM_21	0.853	
ITEM_22	0.847	
ITEM_23	0.833	
ITEM_24	0.804	
ITEM_25	0.838	
ITEM_26	0.818	
ITEM_27	0.834	
ITEM_28	0.846	
ITEM_29	0.847	
ITEM_32		0.682
ITEM_33		0.776
ITEM_34		0.790
ITEM_35		0.849
ITEM_36		0.865
ITEM_37		0.886
ITEM_38		0.867
ITEM_39		0.865
ITEM_40		0.890
ITEM_41		0.877

**FIGURE 1:**
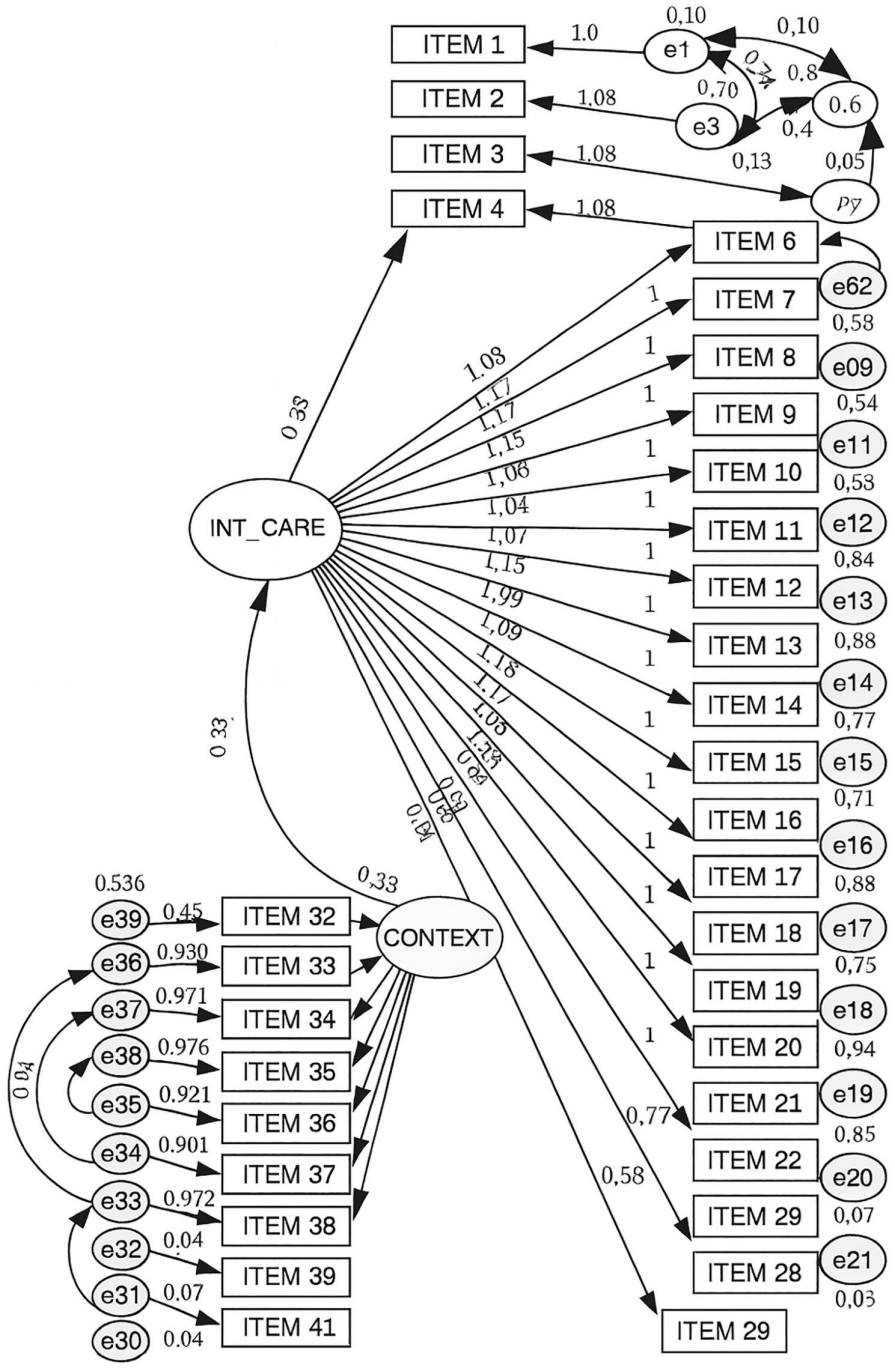
Confirmatory factor analysis.

For the study of construct validity, a CFA was carried out, which yielded the following values: NFI = 0.900; TLI = 0.901; CFI = 0.905; RFI = 0.90; IFI = 0.906; and RMSEA = 0.0789 (Figure 1).

Together, the EFA and CFA findings demonstrate robust construct validity, confirming that the two‐factor model is both statistically sound and conceptually grounded in the FoC Framework.

### 3.3 Predictive Model

**FIGURE 2:**
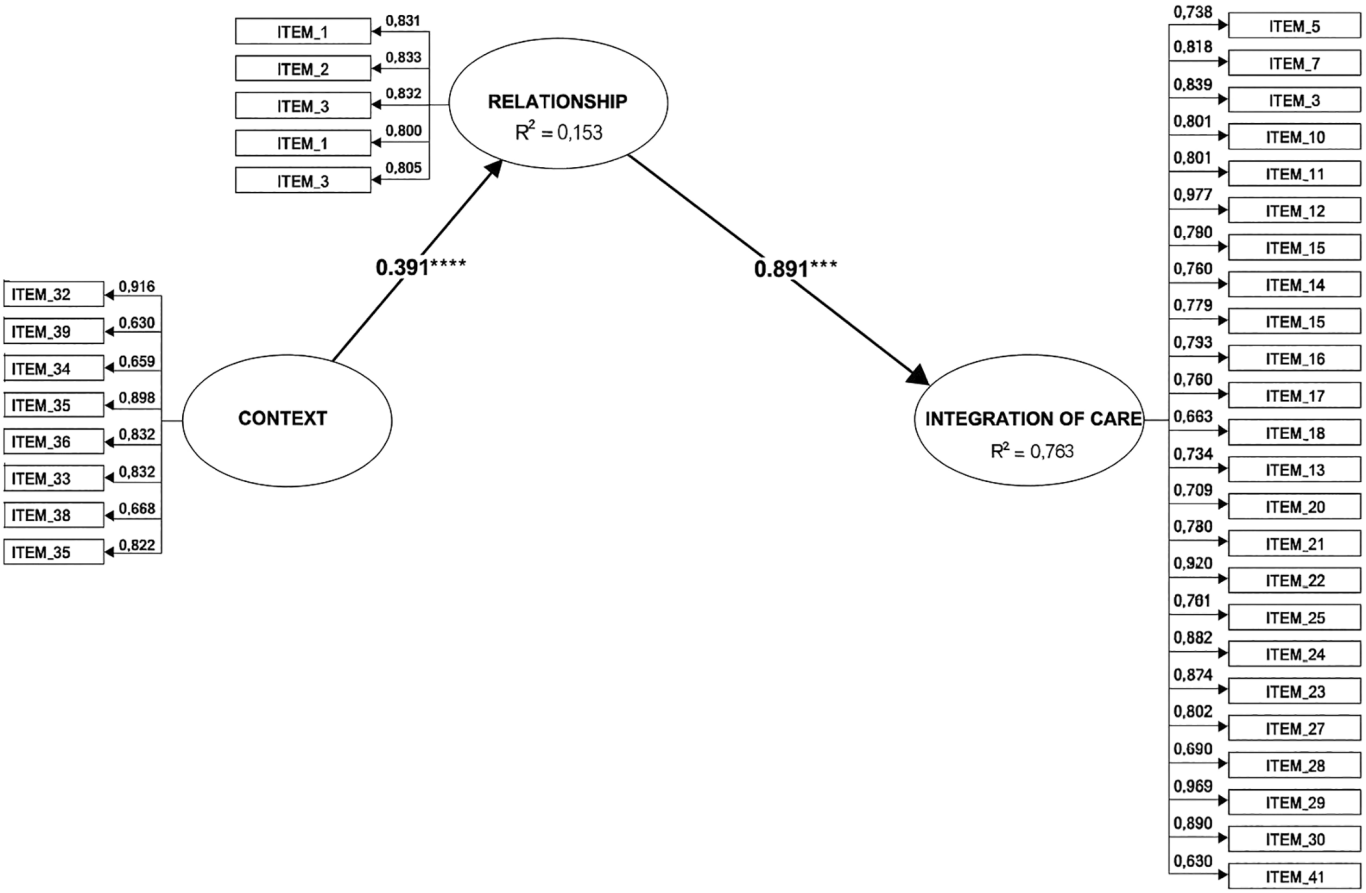
Predictive model of FoC Framework from the patient's perspective.

After identifying the theoretical relationships between the three dimensions—hereafter referred to as composites, following the requirements of the PLS‐SEM approach—of the FoC Framework, and considering the hypotheses described for the present study, a mediation analysis was performed using PLS‐SEM to examine whether the effect of Context on Integration of Care was mediated by Relationship, and to test the hypothesised model. The 39 items extracted by EFA were included in the initial parameter calculation with PLS, although items 40 and 41 were subsequently removed from the model due to collinearity, which occurs when two or more items are highly correlated and therefore provide redundant information, potentially distorting regression estimates (Figure 2).

The values of the fit parameters provided by the PLS method were generally significant (*p* < 0.05): SRMR 0.035 (critical value < 0.08) and AVE between 0.70 and 0.80 (critical value ≥ 0.5). Discriminant validity was confirmed with HTMT values between 0.39 and 0.85 (critical value ≤ 0.85). The VIF values were all between 1.9 and 5 (critical value ≤ 5), demonstrating the lack of collinearity between the 27 items. Reliability analysis obtained values between 0.95 and 0.98 for the parameters Rho A, Cronbach's *α* and Composite Reliability (critical value > 0.7). Additionally, the effect sizes of the relationships between the composites were strong between the Relationship and Integration of Care with *f* ^2^ > 0.35 (critical value > 0.35), and moderate between the Context and Relationship with *f* ^2^ = 0.18 (critical value > 0.15). Concerning the weights of each item in its composite, all of them were between 0.7 and 0.9. In the specific case of each composite, the *R* ^2^ values (see Figure 2, with values inserted in each composite) were between 0.15 and 0.79, indicating the levels of variance explained by the composite.

Regarding the beta coefficients (in Figure 2, values are described in the paths between composites), both are statistically significant (*p* < 0.01). The results of the PLS regression analysis indicate that improvements in the Context composite are associated with improvements in the Relationship composite, with a standardised beta coefficient of 0.391. This means that higher Context scores tend to predict higher scores in Relationship. Similarly, the Relationship composite significantly predicts the Integration of Care composite, with a standardised beta coefficient of 0.891, suggesting that stronger interpersonal relationships are strongly associated with greater integration of care. These results indicate that Relationship acts as a mediator between Context and Integration of Care within the model.

## 4 Discussion

This study validated the Spanish version of the *FoC‐IMT* and revealed important relationships between Context, Relationship and Integration of Care. This aligns with the foundational assumption of the FoC Framework: that caregiving quality is shaped not only by tasks or procedures but by the broader environment and interpersonal relationships in which care occurs (Feo et al. 2017; Kitson 2018).

It is important to note that CFA and PLS‐SEM serve different purposes. CFA, based on covariance, is used to confirm the factorial structure, while PLS‐SEM, based on variances, is used to design predictive models. CFA confirmed that we have two factors: Integration of Care and Context. This means that the data support the existence of these two distinct dimensions within the FoC‐IMT Tool: *context* and *integration of care*. While the original *FoC Framework* delineates *relationship* as a distinct and foundational dimension (Kitson 2018), the present findings suggest that, from the patient's perspective, relational aspects may be experienced as an inherent component of care integration (Langberg et al. 2019). This empirical convergence may stem from the way trust, empathy and therapeutic communication are operationalised in clinical practice, not as isolated processes but as integral features of how care is coordinated and delivered.

In contrast, PLS‐SEM enabled a more nuanced exploration of the interrelationships among the three original dimensions of the *FoC Framework*, which is a distinctive strength of this statistical approach. This analysis confirmed the unique and pivotal role of the Relationship composite, demonstrating that it should not be subsumed under Integration of Care. Specifically, the results revealed that Relationship exerts a strong and significant influence on Integration of Care, and functions as a mediator in the structural model: Context impacts Relationship, which in turn significantly predicts Integration of Care. This mediation analysis confirmed that Relationship mediates the effect of Context on Integration of Care, highlighting its pivotal role as an enabling mechanism within the FoC Framework. In this sense, our findings similarly affirm that Relationship is not simply an affective by‐product of good care but a powerful enabler of Integration of Care, because PLS‐SEM revealed that Context significantly influences Relationship (*β* = 0.39), which in turn strongly predicts Integration of Care (*β* = 0.89).

While the CFA results supported a robust two‐factor structure, PLS‐SEM provided additional insights by modelling the three original theoretical dimensions of the FoC Framework as composites and exploring their interrelationships. Rather than contradicting each other, these methods are complementary (Voss 2023): CFA confirms the factorial validity and parsimony of the measurement model, whereas PLS‐SEM enables the testing of more complex structural relationships, including mediation pathways, that reflect the theoretical underpinnings of the framework. By applying both approaches, this study contributes to a more comprehensive validation of the instrument. CFA establishes empirical rigour in the dimensionality of patient‐reported data, while PLS‐SEM demonstrates how the theoretical constructs interact within the care process.

On the other hand, feedback from PREMs has shown that patients who feel their nurses are attentive, respectful and supportive report greater satisfaction and increased adherence to care plans (Bull et al. 2022; Shunmuga Sundaram et al. 2022). While PREMs offer essential insights into how care is delivered from the patient's perspective, their scope is typically limited to perceptions and experiences rather than measurable outcomes. However, once experience‐based constructs are captured using a psychometrically validated instrument, such as the Spanish version of *FoC‐IMT*, they can also serve as PROMs (Lowry et al. 2024). Importantly, PROMs grounded in patient experience allow for the quantification of key aspects of care quality and their impact on broader health outcomes. As such, the instrument not only reflects how patients perceive the care they receive but also becomes a reliable metric to inform service improvement, evaluate health system performance and guide policy development in person‐centred care (Benson 2023).

### 4.1 Implications for Clinical Practice

The validated factorial structure of the *FoC‐IMT* provides clinicians with a concise, psychometrically robust instrument for capturing patients' perceptions of care provided under the FoC Framework. The predictive model validated further highlights the *Relationship* composite as a mediating construct, indicating that therapeutic engagement between nurses and patients plays a pivotal role in how care environments and organisational processes are perceived and experienced.

From a clinical governance perspective, this underscores the need to prioritise relationship‐building as a measurable and actionable quality indicator, integrated into staff training, reflective practice and patient feedback mechanisms. The value of improving working relationships in multidisciplinary teams has recently been demonstrated in a meta‐analysis in terms of real patient outcomes, showing that the risk of patient death can be reduced by 28% (Webster et al. 2024).

Finally, the use of the *FoC‐IMT* as both a PREM and, in validated form, as a PROM, enables healthcare providers to move beyond anecdotal feedback, systematically evaluating the impact of care processes on patient outcomes, engagement and satisfaction.

The findings encourage the integration of relational metrics into broader quality improvement strategies, thereby supporting the delivery of care that is not only clinically effective but also contextually responsive and person‐centred.

### 4.2 Implications for Future Research

These findings represent a preliminary yet promising step in the refinement and empirical validation of the FoC framework. Further research is required to confirm the stability and generalisability of the factorial structure, using EFA and CFA analyses, and predictive relationships identified in this study, using PLS‐SEM. Extending this work through rigorous cross‐cultural and cross‐contextual analyses may contribute to the development of a broader suite of validated instruments that more accurately reflect the complexity of patient care experiences and support the routine integration of the FoC framework in diverse clinical settings.

In addition, by operationalising the FoC Framework through a quantitative measure of patient experience, this study could also contribute to the development of a middle‐range theory in nursing. Middle‐range theories, which bridge abstract conceptual models and observable clinical practice, are instrumental in advancing disciplinary knowledge while remaining grounded in the realities of person‐centred care (Leandro et al. 2020). Nevertheless, substantial further empirical evidence is still required to robustly support this theoretical development and to confirm its relevance across diverse care settings.

### 4.3 Strengths and Limitations

This study has several strengths that enhance its methodological and theoretical contribution. Chief among them is the dual application of covariance‐based (EFA and CFA) and variance‐based (PLS‐SEM) approaches, which provided complementary insights into both the internal structure of the *FoC‐IMT* and the predictive relationships among the three core dimensions of the FoC Framework. Additionally, the tool was developed and validated using a large sample of hospitalised patients in Spain, supporting its contextual relevance and responsiveness to patient experience in this country.

Nonetheless, several limitations warrant careful consideration. The cross‐sectional design limits causal inference and precludes longitudinal analysis of change over time. Although data collection was interviewer‐administered, which could introduce social desirability bias, rigorous training protocols and standardised procedures were applied to minimise this risk. Likewise, the validation of the tool only in Spain limits its use in other population contexts, without a prior process of translation and cultural adaptation.

## 5 Conclusions

The present study successfully conducted a psychometric validation of the Spanish version of the FoC‐IMT Tool. The comprehensive analysis encompassed both exploratory and confirmatory factor analyses, coupled with the development of a predictive model using partial least squares regression. The findings underscore the pivotal role of the Context of Care in enhancing the Relationship dimension, which, in turn, significantly impacts the Integration of Care.

This tripartite interaction underscores the crucial role of contextual factors in shaping effective caregiver–patient relationships, thereby ensuring that individuals' fundamental physical and psychosocial needs are adequately addressed.

The results demonstrate that the Spanish *FoC‐IMT* is a reliable and valid instrument for assessing the quality of care within hospital settings. The significant beta coefficients observed between the core dimensions of the FoC Framework underscore the robustness of the theoretical framework, confirming the critical influence of the context and the relational aspect in the integration of care practices. These insights not only validate the structural integrity of the FoC framework but also provide a solid foundation for future research aimed at improving care delivery models.

## Author Contributions

All authors made substantial contributions to conception and design or acquisition of data or analysis and interpretation of data; involved in drafting the manuscript or revising it critically for important intellectual content; given final approval of the version to be published; agreed to be accountable for all aspects of the work in ensuring that questions related to the accuracy or integrity of any part of the work are appropriately investigated and resolved. Each author should have participated sufficiently in the work to take public responsibility for appropriate portions of the content.

## Ethics Statement

Ethical approval was secured from relevant bodies, ensuring all procedures adhered to the Declaration of Helsinki principles: *Andalusian government's Ethics Committee*: Approval Code 0840‐N‐22, issued in 2022.

## Consent

All participants provided informed consent, ensuring ethical compliance throughout the study.

## Conflicts of Interest

The authors declare no conflicts of interest.

## Supporting information


**Data S1:** jan70335‐sup‐0001‐Supinfo.docx.

## Data Availability

The data are available upon request to the corresponding author: Prof. Regina Allande‐Cussó, e‐mail: rallande@us.es.
