# Supplementary material for: How Context Shapes Person‐Centred Fundamental Care Through Nurse–Patient Relationships: Validation of the FoC Intelligence Modelling Tool and Predictive Pathway Analysis
Source: J Adv Nurs. 2025 Nov 11;82(7):7200–10. doi: 10.1111/jan.70335 (PMC13267454; doi:10.1111/jan.70335)
Supplement: Supplementary file 1 — Data S1: jan70335‐sup‐0001‐Supinfo.docx. [file JAN-82-7200-s001.docx]

**Supplementary material**

1. **English: Australian FoC Intelligence Modelling Tool**

Relationship Dimension

Please indicate how often the health personnel does any of these:

| Activity | 1 Never | 2 Sometimes | 3 Often | 4 Many times | 5 Always |
| --- | --- | --- | --- | --- | --- |
| Created a climate of mutual trust in which you can express yourself freely and feel safe to do so |  |  |  |  |  |
| Focused attention on you and your health condition |  |  |  |  |  |
| Anticipated your needs by identifying them and trying to respond to them |  |  |  |  |  |
| Showed interest in knowing your health history, personal history, and other relevant information related to your health condition |  |  |  |  |  |
| Evaluated the achievements and final outcome of your visit or health care process and conveyed this information to you |  |  |  |  |  |

Integration of Care Dimension: Physical - Care Recipient Needs

Please indicate how often the health personnel does any of these:

| Activity | 1 Never | 2 Sometimes | 3 Often | 4 Many times | 5 Always |
| --- | --- | --- | --- | --- | --- |
| Took into account your basic needs (eating, drinking, hygiene, dressing, mobility), helped you with them, and respected your wishes |  |  |  |  |  |
| Assisted you with medication management or therapeutic regimens |  |  |  |  |  |
| Made it easier for you to feel comfortable |  |  |  |  |  |
| Made it easier for you to feel safe |  |  |  |  |  |
| Showed interest in your mobility problems and helped you cope with them, if necessary |  |  |  |  |  |

Integration of Care: Psychosocial - Care Recipient Needs

Please indicate how often the health personnel does any of these:

| Activity | 1 Never | 2 Sometimes | 3 Often | 4 Many times | 5 Always |
| --- | --- | --- | --- | --- | --- |
| Allowed and made it easy for you to ask questions about your health situation |  |  |  |  |  |
| Respected your rhythm and time when speaking |  |  |  |  |  |
| Made it easier for you to find meaning or understand your health situation |  |  |  |  |  |
| Helped you feel good |  |  |  |  |  |
| Helped you to recover or attain certain emotional and physical balance |  |  |  |  |  |
| Considered your spiritual needs, values, and beliefs |  |  |  |  |  |
| Showed respect for you |  |  |  |  |  |
| Made it easy for you to get involved in the care you need and provided enough information about your health condition |  |  |  |  |  |
| Provided you with sufficient information and knowledge about your health condition |  |  |  |  |  |
| Provided care in a dignified manner and with sufficient privacy |  |  |  |  |  |

Integration of Care: Relational - Caregiver Actions

Please indicate how often the health personnel does any of these:

| Activity | 1 Never | 2 Sometimes | 3 Often | 4 Many times | 5 Always |
| --- | --- | --- | --- | --- | --- |
| Demonstrated that she/he will be available if you need her/him |  |  |  |  |  |
| Helped you find motivation to improve your health situation or feel better |  |  |  |  |  |
| Helped you set realistic goals regarding your health condition |  |  |  |  |  |
| Helped you cope with stress or distress |  |  |  |  |  |
| Shown empathy, putting themselves in your shoes |  |  |  |  |  |
| Helped you to feel calm |  |  |  |  |  |
| Demonstrated active listening |  |  |  |  |  |
| Has been engaged in helping you |  |  |  |  |  |
| Has been compassionate: identified your suffering and initiated actions to alleviate it |  |  |  |  |  |

Context Dimension

Please indicate your level of agreement with the following statements:

| Statement | 1 Not at all | 2 Slightly | 3 Moderately | 4 Very | 5 Extremely |
| --- | --- | --- | --- | --- | --- |
| This center has sufficient equipment and materials |  |  |  |  |  |
| There are enough staff to treat you |  |  |  |  |  |
| The physical environment facilitates care |  |  |  |  |  |
| This center respects your cultural aspects and values |  |  |  |  |  |
| All staff introduced themselves with their name and position |  |  |  |  |  |
| Each staff member knows their specific function |  |  |  |  |  |
| The staff team is supervised and coordinated appropriately |  |  |  |  |  |
| The centre provides ongoing education and training to its professionals |  |  |  |  |  |
| The staff works together as a team |  |  |  |  |  |
| The center receives support for research projects to improve quality of care |  |  |  |  |  |
| The centre has undergone quality and safety evaluation processes |  |  |  |  |  |
| The center meets the objectives and results expected by Australian Health System or Government |  |  |  |  |  |

1. **Spanish FoC Intelligence Modelling Tool**

Dimensión de Relación (5 Ítems)

Califique las siguientes declaraciones sobre el personal de salud que lo atendió utilizando la siguiente escala:

| **Escala de calificación** | **1 - Nunca** | **2 - A veces** | **3 - A menudo** | **4 - Muchas veces** | **5 - Siempre** |
| --- | --- | --- | --- | --- | --- |
| Ha creado un clima de confianza mutua, permitiéndole expresarse libremente. |  |  |  |  |  |
| Ha centrado su atención en usted y en su estado de salud. |  |  |  |  |  |
| Ha anticipado sus necesidades, identificándolas y tratando de responder a ellas. |  |  |  |  |  |
| Ha mostrado interés en conocer su historial de salud, antecedentes personales y otra información relevante. |  |  |  |  |  |
| Ha evaluado los logros durante el ingreso hospitalario y le ha transmitido esta información. |  |  |  |  |  |

Integración de la Atención: Física - Necesidades del Receptor de la Atención (24 Ítems)

Califique las siguientes declaraciones sobre el personal de salud que lo atendió utilizando la siguiente escala:

| **Escala de calificación** | **1 - Nunca** | **2 - A veces** | **3 - A menudo** | **4 - Muchas veces** | **5 - Siempre** |
| --- | --- | --- | --- | --- | --- |
| Ha tenido en cuenta sus necesidades básicas (comer, beber, higiene, vestirse, movilidad), le ha ayudado con ellas, respetando sus deseos y preferencias. |  |  |  |  |  |
| Le ha ayudado con el manejo de medicamentos o regímenes terapéuticos prescritos por los médicos (por ejemplo, administrándole la medicación, explicándole cómo tomarla, ayudándole con ejercicios prescritos, etc.). |  |  |  |  |  |
| Ha hecho que sea más fácil para usted sentirse cómoda/o. |  |  |  |  |  |
| Ha hecho que fuera más fácil para usted sentirse segura/o. |  |  |  |  |  |
| Ha mostrado interés en sus problemas de movilidad o autocuidado (higiene, alimentación, uso del baño) y le ha ayudado a sobrellevarlos. |  |  |  |  |  |

Integración de la Atención: Psicosocial - Necesidades del Receptor de la Atención

Califique las siguientes declaraciones sobre el personal de salud que lo atendió utilizando la siguiente escala:

| **Escala de calificación** | **1 - Nunca** | **2 - A veces** | **3 - A menudo** | **4 - Muchas veces** | **5 - Siempre** |
| --- | --- | --- | --- | --- | --- |
| Le ha permitido y facilitado hacer preguntas sobre su situación de salud. |  |  |  |  |  |
| Ha respetado su ritmo y tiempo cuando usted hablaba. |  |  |  |  |  |
| Le ha ayudado a comprender su situación de salud. |  |  |  |  |  |
| Le ha ayudado a sentirse bien. |  |  |  |  |  |
| Le ha ayudado a recuperar o alcanzar cierto equilibrio emocional y físico. |  |  |  |  |  |
| Ha considerado sus necesidades espirituales, valores y creencias. |  |  |  |  |  |
| Ha sido respetuosa/o con usted. |  |  |  |  |  |
| Le ha facilitado la participación en los cuidados. |  |  |  |  |  |
| Le ha proporcionado suficiente información acerca de su estado de salud y los cuidados aplicados. |  |  |  |  |  |
| Le ha prestado atención y cuidado de manera digna y con suficiente privacidad. |  |  |  |  |  |

Integración del Cuidado: Acción Relacional – Cuidador

Califique las siguientes declaraciones sobre el personal de salud que lo atendió utilizando la siguiente escala:

| **Escala de calificación** | **1 - Nunca** | **2 - A veces** | **3 - A menudo** | **4 - Muchas veces** | **5 - Siempre** |
| --- | --- | --- | --- | --- | --- |
| Le ha demostrado que estará disponible si usted lo necesita. |  |  |  |  |  |
| Le ha ayudado a encontrar motivación para mejorar su situación de salud o sentirse mejor. |  |  |  |  |  |
| Le ha ayudado a establecer metas realistas con respecto a su estado de salud. |  |  |  |  |  |
| Le ha ayudado a afrontar el estrés o la angustia. |  |  |  |  |  |
| Ha mostrado empatía, poniéndose en su lugar para comprender mejor su situación. |  |  |  |  |  |
| Le ha ayudado a sentirse tranquilo/a. |  |  |  |  |  |
| Le ha escuchado de forma activa. |  |  |  |  |  |
| Le ha demostrado que quería ayudarle. |  |  |  |  |  |
| Ha sido compasivo/a con usted: ha identificado su sufrimiento e iniciado acciones para aliviarlo. |  |  |  |  |  |

Dimensión del Contexto (12 Ítems)

Por favor, califique su nivel de acuerdo con respecto a las siguientes declaraciones sobre el CENTRO DE SALUD y SU FUNCIONAMIENTO/GESTIÓN utilizando la siguiente escala:

| **Escala de calificación** | **1 - En absoluto** | **2 - Ligeramente** | **3 - Moderado** | **4 - Muy** | **5 - Extremadamente** |
| --- | --- | --- | --- | --- | --- |
| El centro cuenta con equipos y materiales suficientes. |  |  |  |  |  |
| Hay suficiente personal para atenderle. |  |  |  |  |  |
| El entorno físico (consultas, despachos, salas de espera, etc.) facilita que el personal pueda atenderle correctamente. |  |  |  |  |  |
| El centro respeta sus aspectos culturales y sus valores. |  |  |  |  |  |
| Todo el personal se presentó con su nombre y su posición o cargo. |  |  |  |  |  |
| Cada miembro del personal tiene una función específica y sabe exactamente lo que debe hacer. |  |  |  |  |  |
| El equipo de personal es supervisado y coordinado por alguien con esa función. |  |  |  |  |  |
| El centro proporciona educación y formación continua a los profesionales. |  |  |  |  |  |
| El personal trabaja en equipo. |  |  |  |  |  |
| El centro recibe suficiente apoyo para implementar proyectos de investigación o mejorar la calidad de la atención. |  |  |  |  |  |
| El centro ha sido objeto de acreditación y evaluación de calidad y seguridad. |  |  |  |  |  |
| El centro debe responder a los objetivos y resultados del Sistema de Salud Nacional. |  |  |  |  |  |
